# Histone deacetylase (HDAC) 1 and 2 are essential for normal T cell development and genomic stability in mice

**DOI:** 10.1186/1868-7083-5-S1-S11

**Published:** 2013-08-19

**Authors:** Oliver M Dovey, George Vasilliou, Allan Bradley, Shaun M Cowley

**Affiliations:** 1 Department of Biochemistry, Henry Wellcome Building, University of Leicester, Leicester, LE1 9HN, UK; 2 The Wellcome Trust Sanger Institute, Wellcome Trust Genome Campus, Hinxton, Cambridge, CB10 1SA, UK

## 

The highly related enzymes, histone deacetylase 1 and 2 (HDAC1/2), regulate chromatin structure as the catalytic core of the Sin3A, NuRD and CoREST co-repressor complexes. To better understand their role in the adaptive immune system and inform their exploitation as drug targets, we have generated mice with a T-cell specific inactivation of both *Hdac1/2* genes. Loss of either HDAC1 or HDAC2 alone has little effect, while dual inactivation results in a 5-fold reduction in thymocyte cellularity, accompanied by developmental arrest at the double-negative to double-positive transition. Mice with reduced HDAC1/2 activity (*Hdac1* deleted and a single *Hdac2* allele) develop a lethal pathology by 3-months of age, caused by neoplastic transformation of immature T cells in the thymus. Tumor cells become aneuploid, express increased levels of c-Myc and show elevated levels of the DNA damage marker, γH2AX. Intriguingly, recent data has shown that these same hypomorphic tumour cells show increased levels of cell death in response to treatment with the HDAC inhibitor, SAHA. Therefore, although a partial reduction in HDAC1/2 activity (>60% of WT) could potentially encourage tumour growth, it may also be an Achilles heel for the treatment of cancer cells with standard HDAC inhibitor therapy.

